# Evaluation of Xpert® MTB/RIF and Ustar EasyNAT™ TB IAD for diagnosis of tuberculous lymphadenitis of children in Tanzania: a prospective descriptive study

**DOI:** 10.1186/s12879-016-1578-z

**Published:** 2016-06-06

**Authors:** Maira Bholla, Neema Kapalata, Edward Masika, Hassan Chande, Levan Jugheli, Mohamed Sasamalo, Tracy R. Glass, Hans-Peter Beck, Klaus Reither

**Affiliations:** Swiss Tropical and Public Health Institute, Socinstrasse 57, 4002 Basel, Switzerland; University of Basel, Petersplatz 1, 4001 Basel, Switzerland; Ifakara Health Institute, P.O. Box 74, Bagamoyo, Tanzania; National TB and Leprosy Clinic, Temeke District Hospital, P.O. Box 45232, Dar es Salaam, Tanzania; Department of Histopathology, Muhimbili University of Health and Allied Sciences, P.O. Box 65000, Dar es Salaam, Tanzania; The Aga Khan Hospital, P.O. Box 2289, Dar es Salaam, Tanzania

**Keywords:** Mycobacterium tuberculosis, Lymphadenitis, Childhood, Fine needle aspiration, Nucleic acid amplification, Xpert MTB/RIF, Ustar EasyNAT, Culture, Cytology, Ziehl-Neelsen

## Abstract

**Background:**

Fine needle aspiration biopsy has become a standard approach for diagnosis of peripheral tuberculous lymphadenitis. The aim of this study was to compare the performance of Xpert MTB/RIF and Ustar EasyNAT TB IAD nucleic acid amplification assays, against acid-fast bacilli microscopy, cytology and mycobacterial culture for the diagnosis of TB lymphadenitis in children from a TB-endemic setting in Tanzania.

**Methods:**

Children of 8 weeks to 16 years of age, suspected of having TB lymphadenitis, were recruited at a district hospital in Tanzania. Fine needle aspirates of lymph nodes were analysed using acid-fast bacilli microscopy, liquid TB culture, cytology, Xpert MTB/RIF and EasyNAT. Latent class analysis and comparison against a composite reference standard comprising “culture and/or cytology” was done, to assess the performance of Xpert MTB/RIF and EasyNAT for the diagnosis of TB lymphadenitis.

**Results:**

Seventy-nine children were recruited; 4 were excluded from analysis. Against a composite reference standard of culture and/or cytology, Xpert MTB/RIF and EasyNAT had a sensitivity and specificity of 58 % and 93 %; and 19 % and 100 % respectively. Relative to latent class definitions, cytology had a sensitivity of 100 % and specificity of 94.7 %.

**Conclusions:**

Combining clinical assessment, cytology and Xpert MTB/RIF may allow for a rapid and accurate diagnosis of childhood TB lymphadenitis. Larger diagnostic evaluation studies are recommended to validate these findings and on Xpert MTB/RIF to assess its use as a solitary initial test for TB lymphadenitis in children.

## Background

Paediatric tuberculosis (TB) contributes considerably to the global burden of TB disease. The World Health Organization (WHO) reported that of the 9.6 million incident cases in 2014, an estimated 10.4 % (1 million) were children [1]. Other groups however, estimated the TB incidence for the same year, as between 200,000 and 970,000 new cases [2–4]. The large variation of those estimates illustrates the difficulties in assessing the disease burden in children, as recording and reporting of paediatric cases is often imperfect and ultimate confirmation of TB diagnosis is still a challenge.

Reasons for the difficulties in diagnosing TB in children are manifold: respiratory specimens from younger children are difficult to obtain, *Mycobacterium tuberculosis* (*M. tuberculosis*) is often not detectable in paucibacillary paediatric specimens and host biomarkers are not yet in place to reliably identify children with TB disease [5, 6]. Moreover, symptoms and radiological features are non-specific and overlap with those of other diseases, such as pneumonia, malnutrition or HIV-associated diseases. The tuberculin skin test (TST), which indicates TB infection, can guide clinicians in their decisions, but does not confirm TB disease [7]. Hence, better diagnostic tests for both pulmonary and extrapulmonary paediatric TB are urgently needed.

Extrapulmonary TB (EPTB) constitutes up to half of all paediatric TB cases, predominantly in children of young age and with HIV infection [8, 9]. The most common manifestation of EPTB in children is tuberculous lymphadenitis (TB lymphadenitis) in about two thirds of EPTB cases, followed by central nervous system involvement (meningitis), pleural, disseminated and skeletal TB [9]. TB lymphadenitis may be mediastinal, intra-abdominal or, as was the focus of this study, peripheral. This form is typically superficial, and often presents as a non-specific swelling which may be mistaken for malignant in nature [10]. Previous studies in East-African adults show that peripheral lymphadenitis is frequently caused by non-tuberculous mycobacteria or *M. bovis* [11, 12]; data on these etiologies in East-African children, however, are still scarce [13, 14].

Fine needle aspiration biopsy (FNAB), an inexpensive, easy-to-perform outpatient procedure, has become the diagnostic approach of choice for children with persistent lymphadenitis and is suited for primary health care [15–17]. Diagnostic microscopy is used for cytology, for smears after Ziehl-Neelsen (ZN) staining, and for autofluorescence detection on Papanicolaou stained smears. If combined, these three modalities have a reasonably high sensitivity and specificity compared to culture [18]. However, neither culture nor microscopy-based diagnostic approaches are considered a gold standard for TB lymphadenitis. These tests are still rarely used in peripheral facilities of resource-poor settings: culture is complex and implicates a high biosafety level; interpretation of cytology requires advanced technical expertise and small laboratories are often not equipped with fluorescence or light-emitting diode (LED) microscopes.

Automated and isothermal nucleic acid amplification tests (NAAT), designed for use at low biosafety levels, could offer promising alternatives for point-of-care diagnosis of TB lymphadenitis.

Xpert® MTB/RIF (Cepheid, Sunnyvale, USA) is an automated, cartridge-based NAAT for the detection of *M. tuberculosis* complex and rifampicin drug-resistance from sputum and other specimens, with a turnaround time of less than two hours [19, 20]. A WHO policy statement from 2013 formulated recommendations to guide the use of the test for pulmonary and extrapulmonary TB in adults and children. Based so far only on very low evidence, Xpert has been conditionally recommended as a replacement test for usual practice (including conventional microscopy, culture or histopathology) for testing specific non-respiratory specimens, including lymph nodes [21].

The EasyNAT™ TB Isothermal Amplification Diagnostic Kit (short form: EasyNAT; Ustar, Biotechnologies Co. Ltd, Hangzhou, China) is an assay that uses isothermal cross-priming amplification technology for the qualitative detection of *M. tuberculosis* [22]. The tool was approved for detection of pulmonary TB by the China Food and Drug Administration in 2014 [23]. Up to now, no studies have been published on EPTB diagnosis using EasyNAT.

The aim of our study was to compare the diagnostic performance of Xpert MTB/RIF and EasyNAT with acid-fast bacilli (AFB) microscopy, cytology and mycobacterial culture for the diagnosis of TB lymphadenitis in children from a TB-endemic setting in Tanzania.

## Methods

### Recruitment and clinical assessment

This prospective study was carried out from April 2012 until August 2013 at the Temeke District Hospital in Dar es Salaam, Tanzania. Children older than 8 weeks and younger than 16 years of age, suspected of having TB lymphadenitis as defined by WHO [24], were recruited. Inclusion criteria were: one or more palpable lymph nodes of 1 cm or more persisting for more than 4 weeks in-spite of oral antibiotic therapy and a strong clinical suspicion or microbiological confirmation of mycobacterial infection. Children who had received TB treatment in the preceding 12 months were excluded from the study. All participants were clinically assessed and treated according to current versions of the Tanzanian National TB and Leprosy Program (NTLP) guidelines [25, 26]. For children up to 6 years of age, the clinical assessment was based on the NTLP clinical scoring chart. Results from the evaluated diagnostic approaches also had an impact on treatment decisions.

At enrolment, clinical assessment comprised chest X-ray in a frontal view, TST, full blood count and HIV testing with pre- and post-test counselling. The HIV rapid testing algorithm followed national guidelines [27]. HIV DNA nucleic acid test was done for children between 8 weeks and 18 months of age, while for older children antibodies to HIV were detected using rapid tests or Enzyme Immunoassays. FNAB was performed on all participants. Expectorated sputa were collected from children with productive cough and sent for AFB microscopy to the district laboratory. At a follow-up visit (time after recruitment: 5–12 months; median 5 months), participants were assessed for treatment outcomes and referred for further treatment, if necessary.

### Palpation-guided fine needle aspiration biopsy

Palpation-guided FNAB was carried out by trained medical officers on palpable regional lymph nodes. FNAB was performed with a 23-gauge needle attached to a 10 mL syringe. The skin around the lymph node was cleaned with an alcohol swab, the child was held firmly and the node was stabilized while aspiration was performed, with not more than 2 mL of suction.

Two smears were prepared from the first aspirate. One smear was immediately fixed in 95 % ethanol solution for Papanicolaou staining for cytological assessment and the other was air-dried and fixed in 100 % methanol for ZN staining. The residue in needle and syringe was rinsed in mycobacterial growth indicator tube (MGIT) medium for mycobacterial culture with standard PANTA antibiotic/growth supplement (Beckton Dickinson, Sparks, USA) [28]. The MGIT tube was transported at ambient temperature to the laboratory for incubation and further processing.

From the same lymph node, a second aspiration was performed and rinsed into 0.7 ml sterile phosphate buffered saline (PBS), pre-prepared in 1.5 ml polypropylene tubes. These were transported to the laboratory at 4-8 °C for NAAT testing. Laboratory personnel involved in Xpert MTB/RIF and EasyNAT testing were blinded to other results.

### Laboratory diagnosis

#### FNA microscopy/cytology

The pathologist reported ZN stained smears as; “AFB seen” or “no AFB seen”. Cytology results were categorised as “confirmed mycobacterial lymphadenitis” (visualization of AFB), “suspected mycobacterial lymphadenitis” (signs of TB necrosis and granuloma consistent with mycobacterial infection), nonspecific “reactive lymphadenopathy” or “suspicion of malignancy” [29].

#### FNA TB culture

The inoculated MGIT tubes were incubated at 37 °C for a maximum of 8 weeks. Positive MGIT cultures were tested for contamination on blood agar. MPT64 antigen and molecular tests as needed (GenoType® MTBDR*plus* system and GenoType MTBC, Hain Lifescience GmbH, Germany) were used for confirmation of *M. tuberculosis* complex and identification of MTBC species, including *M. bovis* BCG. Drug susceptibility testing (DST) was done as described in the BACTEC MGIT 960 product insert (Becton Dickinson) [30]. Non-tuberculous mycobacteria (NTM) were assessed using a reverse line-probe assay (GenoType® Mycobacterium CM/AS, Germany).

#### FNA Xpert MTB/RIF testing

One half of the aspirate in PBS (0.2 to 0.7 ml) per participant was filled up to 2.2 ml with Xpert MTB/RIF sample reagent and incubated for 15 min, prior to dispensing 2 ml of the mixture into a Xpert MTB/RIF. The cartridge was loaded into the Xpert MTB/RIF instrument for automated sample and real-time polymerase chain reaction (RT-PCR) processing. The instrument reported the presence or absence of *M. tuberculosis* complex, a semiquantitative estimate of *M. tuberculosis* complex concentration (high, medium, low, and very low) and the presence or absence of rifampicin resistance.

#### FNA EasyNAT testing

The other half of the aspirate in PBS was processed according to the EasyNAT testing protocol for specimens other than sputum [31]. Very viscous or purulent specimens were first treated with liquefying buffer provided, according to the protocol for sputum specimens. The DNA extracted for each sample was then prepared for amplification and incubated at 63 °C for 60 min [31]. Detection of amplified products was performed on a lateral flow strip, housed in an enclosed sealed plastic device, to prevent leakage of amplicons. Results were visualized after 20 to 30 min. Appearance of the control band alone indicated that the test was valid but that there was no *M. tuberculosis*, or that the amount of *M. tuberculosis* DNA was below the detection limit. In conjunction with a visible control band, positivity was determined by the intensity of the test band in relation to an intensity scale provided [31].

### Statistical analysis

Sensitivity, specificity and Cohen’s Kappa [32] were calculated for each and for all diagnostic modalities combined with culture and cytology as single reference standards, and with culture and cytology combined (*M. tuberculosis* positive when either was positive and *M. tuberculosis* negative when both were negative), as a composite reference standard (CRS). The calculations were performed only for patients without missing data for the respective modalities. Kappa (k) of more than 0.6 indicated substantial to almost-perfect agreement with the reference standard. Latent class analysis (LCA), a statistical method for finding subtypes of related (latent) cases from multivariate categorical data, was performed on all the FNAB diagnostic outcomes. For each diagnostic modality, two classes were identified, 1 for TB positive and 2 for TB negative. Blank cells indicated inconclusive outcomes or missing data. The LCA provided estimates of the probability of *M. tuberculosis*-presence or *M. tuberculosis*-absence per case, based on the data input. Additionally, a mixed logistic regression model with a 5-dimensional binary outcome defined by the results of the 5 test modalities was applied. The model included a random subject intercept and assigned each subject the empirical Bayes estimate of this random effect as a TB-score, which was then correlated with each binary test. Analyses were done using SAS version 9.2 (SAS Institute Inc., Cary, NC, USA) and Stata version 12.1 (StataCorp, College Station, Texas, USA).

## Results

**Fig. 1:**
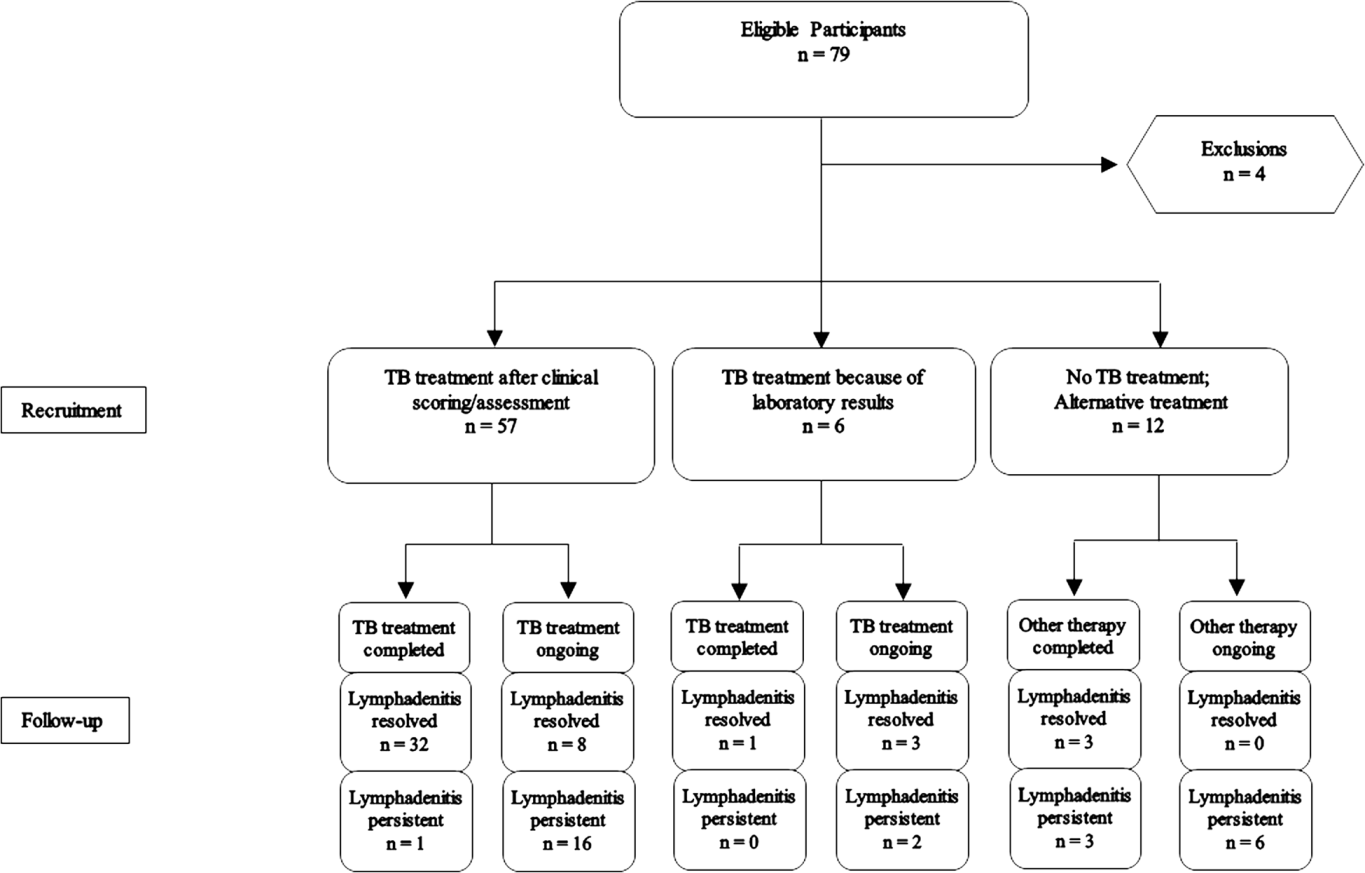
Patient enrolment flow chart and clinical outcomes

A total of 79 children were recruited into this study; 4 children were excluded from analysis because of incomplete data due to death after recruitment, loss of contact, relocation and subsiding lymph node more than 4 weeks after general antibiotic treatment. During the recruitment phase, a standard 6-month anti-TB regimen was started in 57 participants based on NTLP paediatric TB scoring (for children <6 years of age) or clinical assessment (for children ≥6 years of age) and in 6 participants based solely on laboratory test results, i.e. cytology, Xpert MTB/RIF or culture. Participants who did not receive TB treatment (*n* = 12) were reviewed by the internal medicine, surgery or oncology departments. At the follow up visit, the lymphadenitis was resolved in 33/34 children who had completed the anti-TB therapy and 3/6 children who completed alternative therapy (surgical intervention, fungal or antibiotic treatment). Figure 1 displays the patient flow and clinical outcomes.

More boys than girls were recruited (61 % vs. 39 %). The HIV prevalence was 20 %. Only 2 participants, 4 and 11 years of age, had a history of TB. At recruitment one of the three children with additional productive cough was found to be sputum AFB positive. Table 1 summarizes the participant characteristics.

**Table 1:** Participant characteristics

Characteristics at recruitment	Children included in study (*N* = 75) *n* (%)
Age (years)	
0 to ≤ 2	14 (19 %)
2 to ≤ 6	30 (40 %)
6 to ≤ 10	16 (21 %)
10 to ≤16	15 (20 %)
Gender	
Male	46 (61 %)
HIV status	
HIV test positive	15 (20 %)
HIV test negative	54 (72 %)
Unknown/missing/not done	6 (8 %)
TB contact in last 12 months	
Yes	18 (24 %)
General clinical condition	
Good	55 (73 %)
Reduced	15 (20 %)
Ill	5 (7 %)

The frequency of results from the different diagnostic techniques on FNAB is shown in Table 2. In summary, cytology results in 18 children (24 %) were consistent with mycobacterial infection including 3 children with ZN-positive lymphadenitis. *M. tuberculosis* was identified in 7 children (9 %) by culture. *Mycobacterium bovis* BCG was isolated from 2 HIV negative children (3 %) who were 3 and 4 months of age. Xpert MT/RIF was positive in 14 (19 %) and EasyNAT in 5 (7 %) participants. There were no invalid Xpert MTB/RIF or EasyNAT results. No resistance was detected by Xpert MTB/RIF or phenotypic DST.

**Table 2:** Frequency of laboratory findings on FNAB

Diagnostic modalities	Children included in study (*N* = 75) *n* (%)
Cytology	
Consistent with mycobacterial infection^a^	18 (24 %)
Reactive lymphadenopathy (negative)	39 (52 %)
Malignancy/suspected malignancy (negative)	4 (5 %)
Unsuitable for analysis	14 (19 %)
Missing	-
Ziehl Neelsen microscopy	
Acid fast bacilli-positive	3 (4 %)
Negative	71 (95 %)
Missing slide	1 (1 %)
Culture	
*M. tuberculosis*	7 (9 %)
*M. bovis* BCG	2 (3 %)
Negative	29 (39 %)
Non-tuberculous mycobacteria (negative)	1 (1 %)
Contaminated	36 (48 %)
Missing	-
Xpert MTB/RIF	
Positive	14 (19 %)
Negative	56 (74 %)
Missing result	5 (7 %)
EasyNAT	
Positive	5 (7 %)
Negative	70 (93 %)
Missing	-

^a^ Consistent with mycobacterial infection’ includes confirmed TB (AFB seen) and/or suspected TB (morphological features consistent with TB infection – caseating necrosis and granuloma)

**Fig. 2:**
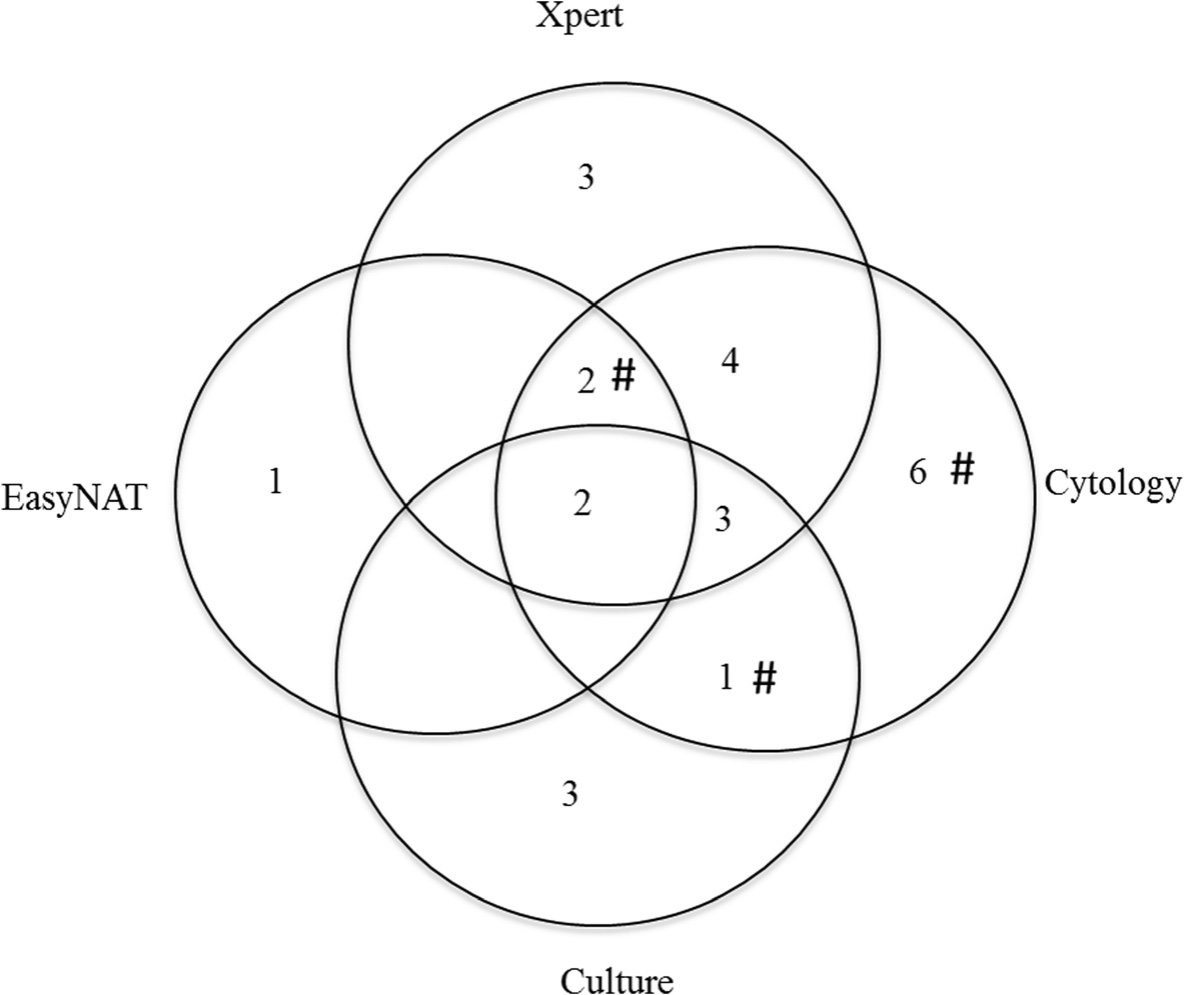
Venn diagram of TB positive results from all diagnostic modalities for FNAB. Diagram shows positive results from culture (including *M. tuberculosis* and *M. bovis* BCG), Cytology, Xpert MTB/RIF and EasyNAT. The 3 AFB positive ZN smears are indicated by #

Figure 2 displays the correlation between the different laboratory testing modalities reporting cases as positive for TB. The culture results comprise both samples with confirmation of *M. tuberculosis* (*n* = 7) and *M. bovis* BCG (*n* = 2). In 25 children, the diagnosis ‘TB lymphadenitis’ was confirmed by at least one of the diagnostic modalities; of which 12 cases were positive by a single diagnostic modality alone.

The performance of each laboratory diagnostic modality was assessed against culture as the reference standard for TB. Due to the low positivity and high contamination rates for culture in this study, cytology was considered as an alternative reference standard. Additionally, a composite reference standard, combining culture and cytology results, was used to assess the performance of the diagnostic tests (Table 3). Xpert MTB/RIF detected TB lymphadenitis with a sensitivity and specificity of 56 and 96 %, 69 and 93 %, 58 and 93 % against a reference standard consisting either of culture, or cytology, or culture and/or cytology respectively. The corresponding sensitivity and specificity values for EasyNAT were 22 and 100 %, 22 and 100 %, and 19 and 100 %. The category “any diagnostic modality” aimed to assess the performance of a diagnostic algorithm including all the modalities except those used as reference standard. The sensitivity of “any diagnostic modality” were either equal or superior compared to single tests, while the specificity of “any diagnostic modality” at 93 % was below specificity values of some single tests.

**Table 3:** Test performance and agreement of diagnostic modalities

	Culture as reference standard					Cytology as reference standard					Composite reference standard: culture and/or cytology				
	Sensitivity^a^		Specificity^a^		Cohen’s Kappa	Sensitivity^a^		Specificity^a^		Cohen’s Kappa	Sensitivity^a^		Specificity^a^		Cohen’s Kappa
	*n/N*		*n/N*		k	*n/N*		*n/N*		k	*n/N*		*n/N*		k
Cytology	6/9	67 %	22/24	92 %	0.60	-	-	-	-	-	-	-	-	-	-
Culture	-	-	-	-	-	6/8	75 %	22/25	88 %	0.60	-	-	-	-	-
ZN	1/9	11 %	24/25	96 %	0.10	3/18	17 %	43/43	100 %	0.22	3/21	14 %	41/41	100 %	0.18
Xpert	5/9	56 %	26/27	96 %	0.58	11/16	69 %	38/41	93 %	0.64	11/19	58 %	40/43	93 %	0.55
EasyNAT	2/9	22 %	30/30	100 %	0.31	4/18	22 %	43/43	100 %	0.29	4/21	19 %	46/46	100 %	0.24
Any diagnostic modality	6/9	67 %	28/30	93 %	0.62	18/18	100 %	40/43	93 %	0.89	13/21	62 %	38/41	93 %	0.58

^a^Calculated only for patients without missing data

The agreement of each diagnostic modality with the appointed reference standard (Cohen’s Kappa – k) is also described in Table 3. Xpert MTB/RIF, compared to EasyNAT, showed a better agreement with TB culture (k = 0.58 vs. 0.31), cytology (k = 0.64 vs. 0.29) and the CRS (k = 0.55 vs. 0.24). ZN microscopy had a poor agreement with the three reference standards.

For LCA two ‘latent classes’ were generated: class 1 for TB-positive (prevalence of TB lymphadenitis in the study population) with a probability of 22.8 % and class 2 for TB-negative with a probability of 77.2 %. The probability of a positive test result given membership to class 1 (i.e., the sensitivity) was high for cytology (100 %), culture (74 %), Xpert MTB/RIF (71 %), but low for EasyNAT (29 %) and ZN (19 %). Conversely, the probability of a negative test result given membership to class 2 (i.e., the specificity) was high for all 5 tests, with 100 % for EasyNAT and ZN, 95 % for Cytology, 94 % for Xpert MTB/RIF and 90 % for culture.

The mixed logistic regression model returned a TB-score for each patient. The correlation of this score with each binary test showed that cytology performed best with a correlation of *r* = 0.87, followed by culture and Xpert MTB/RIF at par (*r* = 0.82), EasyNAT (*r* = 0.60) and ZN (*r* = 0.42).

Positive laboratory test results were analysed in relation to results of the paediatric score chart for children below 6 years of age and to clinical assessment for the older children (Table 4). By assessing the agreement to score or clinical assessment, the true-positive rates for all of the five modalities ranged between 5 and 26 % and false-positive rates between 8 and 33 %.

**Table 4:** FNAB laboratory diagnoses compared to clinical assessment

		Paediatric score <6 years of age (*n* = 44)		Clinical assessment ≥6 years of age (*n* = 31)	
		Score ≥7 (anti-TB therapy recommended)	Score <7 (anti-TB therapy *not* recommended)	Anti-TB therapy recommended	anti-TB therapy *not* recommended
	*n*	*n* = 38	*n* = 6	*n* = 19	*n* = 12
Positive culture	9	5 (13 %)	1 (17 %)	3 (16 %)	-
Positive cytology	18	9 (24 %)	2 (33 %)	5 (26 %)	2 (17 %)
Positive Xpert	14	5 (13 %)	2 (33 %)	5 (26 %)	2 (17 %)
Positive EasyNAT	5	2 (5 %)	-	2 (11 %)	1 (8 %)
Positive ZN	3	2 (5 %)	-	1 (5 %)	-

Percentages in columns for ‘anti-TB therapy recommended’ are true-positive rates

Percentages in columns for ‘anti-TB therapy *not* recommended’ are false-positive rates

## Discussion

We assessed the performance of Xpert MTB/RIF and of EasyNAT for the diagnosis of paediatric TB lymphadenitis, using culture or cytology as single reference standards and a combination of both as a composite reference standard. The results differed considerably between the two NAATs under evaluation. Xpert MTB/RIF performed with a modest sensitivity and good specificity, while the sensitivity of EasyNAT was comparatively poor, with excellent specificity. Both Xpert MTB/RIF and EasyNAT may have been negative in some cases of lymphadenitis caused by NTM infection, which is frequently seen in East Africa [11], but might be undetected in our study due to the high proportion of contaminated cultures. These results were consistent with a Cohen’s kappa statistic, which showed a superior agreement of Xpert MTB/RIF with TB culture, cytology and the CRS compared to EasyNAT.

As an alternative to the CRS approach, a latent class analysis was applied to estimate the sensitivity and specificity of diagnostic tests by linking multiple test results to the same latent categories. This latent class model was used because of the imperfect or missing reference standard [33]. The LCA revealed superior sensitivity estimates for Xpert MTB/RIF compared to EasyNAT (71 % versus 29 %). Conversely, the specificity estimates for EasyNAT were to some extent higher than those for Xpert MTB/RIF (100 % versus 94 %). Interestingly, LCA indicated an excellent sensitivity (100 %) and good specificity (95 %) for cytology.

A systematic review and meta-analysis of 13 studies described a pooled sensitivity and specificity of 83.1 and 93.6 % for the Xpert MTB/RIF assay with TB culture as a reference standard for the diagnosis of lymph node TB [34]. This publication ultimately informed WHO policy and has led to recommendations on the use of Xpert MTB/RIF, over established tests, for diagnosis of TB in lymph nodes in adults and children [35]. An additional meta-analysis which also examined Xpert MTB/RIF against TB culture for the diagnosis of TB lymphadenitis showed a slightly better pooled sensitivity (87 %) and a similar specificity (92 %) [36]. A hospital-based South African FNAB study in 72 children by *Coetzee* et al. reported a sensitivity of 80 % and a specificity of 93.8 % against a CRS (cytology suggestive of mycobacterial disease with visualization of the bacillus and/or positive TB culture) [37]. In contrast to mentioned meta-analyses which focused primarily on adults and the evaluation on children from South Africa, our Xpert MTB/RIF study in children showed substantially lower estimates for sensitivity, but similar estimates for specificity. The limited sensitivity of Xpert MTB/RIF is most likely caused by the paucibacillary nature of lymphatic TB manifestations in children [38] or by suboptimal rinsing of needle passes [37].

The findings underscore the need for larger and representative paediatric accuracy studies for the diagnosis of mycobacterial lymphadenitis by Xpert MTB/RIF and other NAATs. In the future, more sensitive, point-of-care NAAT, which are also able to perform resistance testing, are required to make an impact over current modalities with regard to mortality caused by paediatric and extrapulmonary TB, as described in a recent modelling study [39].

Until now, EasyNAT, a manual assay based on isothermal cross-priming amplification, has only been evaluated for the detection of pulmonary TB in adults [40, 41]. These two studies reported sensitivity for *M. tuberculosis* detection against culture of 84.1 and 66.7 %, and specificity of 97.8 and 100 %. Sensitivity in these studies was further reduced when only smear-negative but culture-positive pulmonary TB cases were tested (59.8 and 10 %) which indicates a suboptimal limit of detection that probably could explain the poor sensitivity with our paediatric patients, where low density of mycobacteria is expected in lymph nodes.

A major limitation of this study was the relatively high proportion of invalid test results; i.e. 19 % of the samples sent for cytology were not suitable for analysis and 48 % of the TB cultures were contaminated. Consequently, the comparison between diagnostic modalities was often restricted to a small number of participants with valid results. The high contamination rate negatively impacted the absolute number of positive TB cultures and therefore reduced the value of TB culture as a reference standard in FNAB.

The greatest promise of NAATs for the detection of TB in lymph nodes of children is fast and accurate diagnosis, combined with simultaneous resistance testing. The incomplete overlap of the different diagnostic modalities suggests that neither Xpert MTB/RIF nor EasyNAT currently seem to be appropriate as a single NAAT to diagnose TB lymphadenitis in children.

## Conclusions

Based on our findings, a promising diagnostic algorithm for suspected childhood TB lymphadenitis seems to consist of the combination of clinical assessment according to WHO guidelines [17], cytology and Xpert MTB/RIF. However, larger studies in children from different settings are needed to prove this diagnostic concept. EasyNAT seems to be not suitable for diagnosis of TB lymphadenitis in children. At high healthcare levels, TB culture might be a valuable method for differentiation between *M. tuberculosis* and *M. bovis* BCG, which is important for management of patients with lymphadenitis, but its broader use is restricted due to the length of time needed to get results, technical complexity, high costs and biosafety requirements.

## Abbreviations

AFB, acid-fast bacilli; BCG, Bacillus Calmette–Guérin; CRS, composite reference standard; DNA, deoxyribonucleic acid; DST, Drug susceptibility testing; EPTB, extrapulmonary tuberculosis; FNAB, fine needle aspiration biopsy; HIV, human immunodeficiency virus; LCA, latent class analysis; LED, light-emitting diode; MGIT, mycobacterial growth indicator tube; NAAT, nucleic acid amplification tests; NIMR, National Institute for Medical Research; NTLP, National TB and Leprosy Program; NTM, non-tuberculous mycobacteria; PBS, phosphate buffered saline; RT-PCR, real-time polymerase chain reaction; Swiss TPH, Swiss Tropical and Public Health Institute; TB, tuberculosis; WHO, World Health Organization; ZN, Ziehl-Neelsen
